# Supplementary material for: Cross-Omic Transcription Factor Analysis: An Insight on Transcription Factor Accessibility and Expression Correlation
Source: Genes (Basel). 2024 Feb 21;15(3):268. doi: 10.3390/genes15030268 (PMC10970009; doi:10.3390/genes15030268)
Supplement: Supplementary file 1 [file genes-15-00268-s001.zip › genes-2851467-supplementary.pdf]

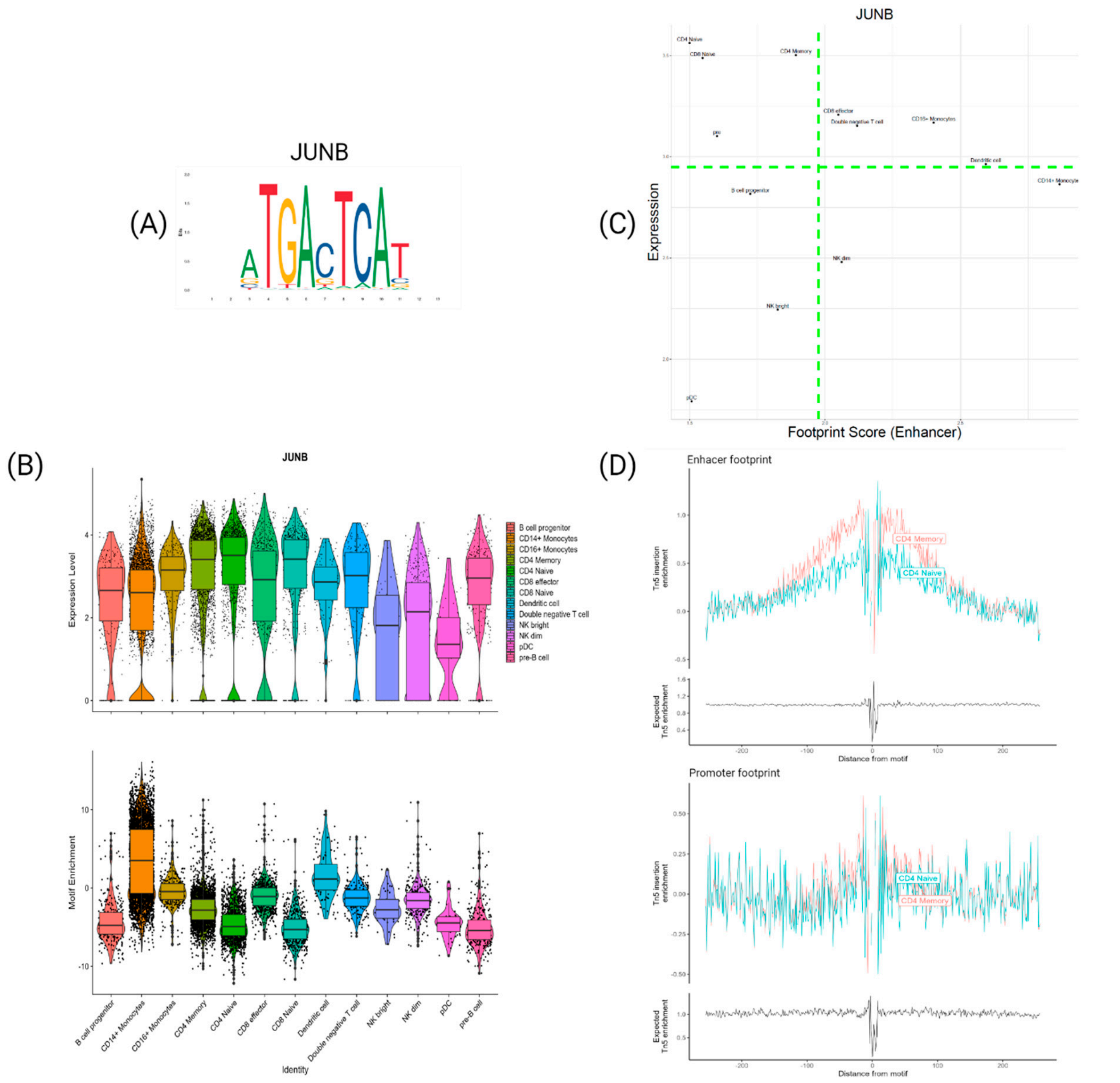

Supplementary Figure S1: Transcription Factor JUNB. **A** PFM visualization for JUNB's motif MA0490.2. **B** Violin plot of expression (top) and motif enrichment (bottom), for all cell types. **C** Scatter plot of footprint score-expression of JUNB for all cell types. **D** Tn5 insertion plots for Memory and Naive CD4 T cells.

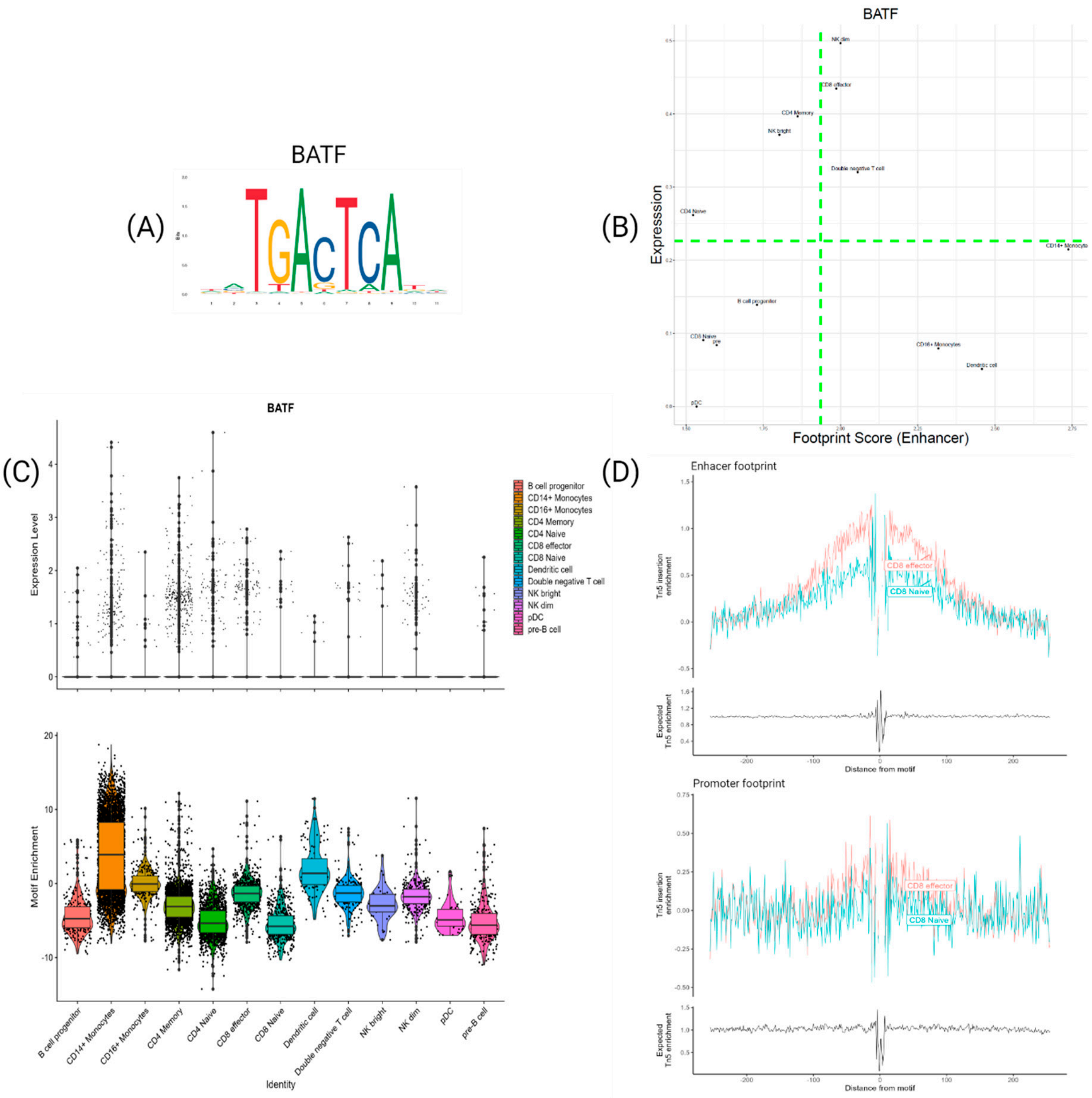

Supplementary Figure S2: Transcription Factor BATF. **A** PFM visualization for BATF 's motif MA1634.1. **B** Violin plot of expression (top) and motif enrichment (bottom), for all cell types. **C** Scatter plot of footprint score-expression of BATF for all cell types. **D** Tn5 insertion plots for Effector and Naive CD8 T cells.

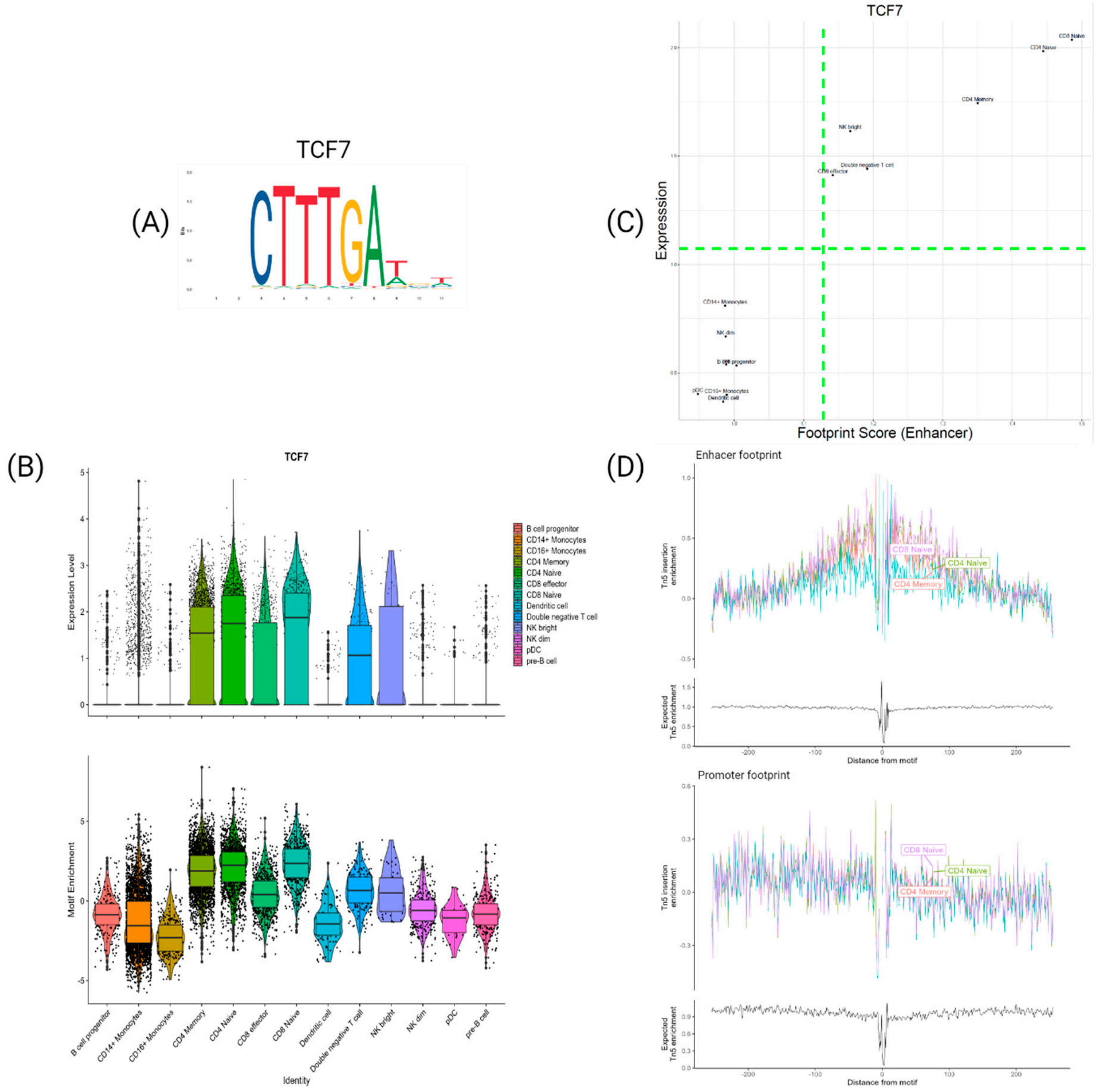

Supplementary Figure S3: Transcription Factor TCF7. **A** PFM visualization for TCF7's motif MA0769.2. **B** Violin plot of expression (top) and motif enrichment (bottom), for all cell types. **C** Scatter plot of footprint score-expression of TCF7 for all cell types. **D** Tn5 insertion plots for Memory and Naive CD4 and Effector and Naive CD8 T cells.

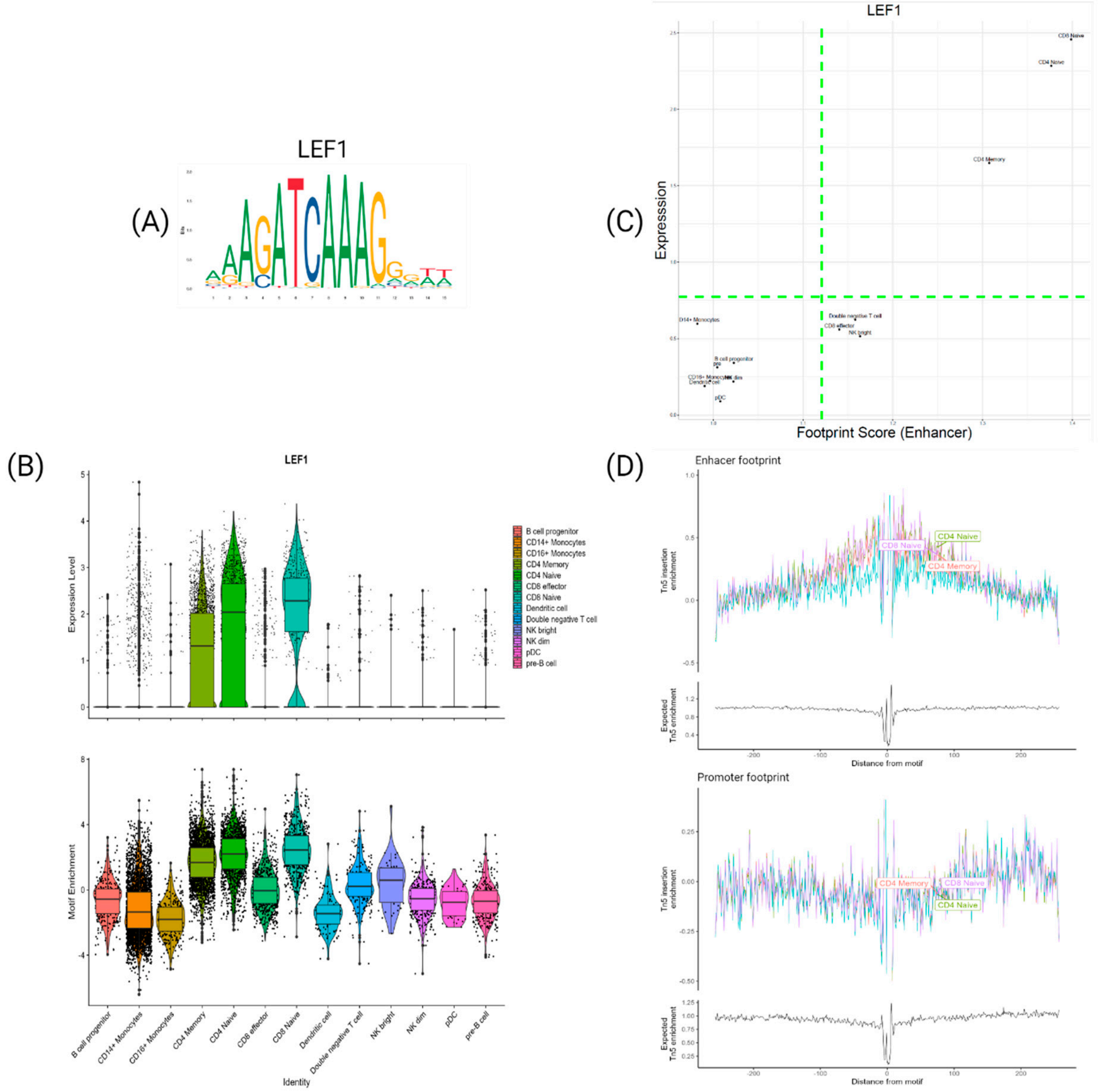

Supplementary Figure S4: Transcription Factor LEF1. **A** PFM visualization for LEF1's motif MA0768.1. **B** Violin plot of expression (top) and motif enrichment (bottom), for all cell types. **C** Scatter plot of footprint score-expression of LEF1 for all cell types. **D** Tn5 insertion plots for Memory and Naive CD4 and Effector and Naive CD8 T cells.
